# Hiding in the Pub to Cutting the Cord? Men’s Presence at Childbirth in Britain *c*.1940s–2000s

**DOI:** 10.1093/shm/hkw057

**Published:** 2016-06-30

**Authors:** Laura King

**Keywords:** childbirth, midwifery, masculinity, fatherhood, Britain

## Abstract

Since the 1940s, men’s presence at childbirth has changed from being out of the question to not only very common but often presented as highly valuable. This article examines this shift, charting how many men were present at their children’s births over recent decades, considering how medical practitioners influenced men’s participation, and analysing what meanings parents gave to this experience. It suggests a number of factors led to the relatively rapid move towards the acceptance of men’s presence in the delivery room, but highlights this was not a simple transformation as a first glance at the figures would suggest. It argues that men’s involvement in home births became more usual before hospitals changed their policies about men’s presence, and considers how the role of fathers related to the increasingly medicalised nature of childbirth as this period progressed. It also considers whether men’s involvement is always positive or welcome for those involved.

## Introduction

In the nineteenth and early-twentieth centuries in Britain, some husbands from aristocratic and elite families were present for their wives’ labours.^1^ This practice became uncommon in the twentieth century, as women increasingly gave birth in medical institutions. Furthermore, this was not usual amongst working-class families.^2^ In the novel (and film) *Love on the Dole*, for example, Harry, about to become a father, is told by a neighbour to ‘Now, lad, sling y’r hook. Y’aint wanted here for hafe an hour an’ y’ll on’y be in way.’^3^ In the film, his entrance to his own house is physically prevented.^4^ Many men and women agreed with this sentiment. By the 1950s and 1960s, there was evidence of gradually changing attitudes and practices, across different social groups. In 1964, a BBC television programme showed footage of a husband accompanying his wife during the birth of their child. This programme, entitled ‘Intimate Union’, suggested that a man’s presence at his wife’s side during labour and delivery reflected and could bring about the more intimate experience of marriage that was said to be increasingly common.^5^

This article focuses on men’s involvement in childbirth, exploring when this change occurred and what the experience of childbirth—and men’s presence in particular—meant for families. It examines social research to ascertain the proportion of men present during childbirth over time; the attitudes of medical practitioners, using original and archived oral history interviews with midwives and doctors, alongside medical literature such as the *British Medical Journal* and *The Lancet*; and parents’ own narratives of childbirth, through original and archived oral history interviews, and an online questionnaire.^6^ Fifty interviews with fathers were conducted from 2013 to 2014, and with archived interviews, these testimonies represent various social backgrounds, generations and parts of Britain. These interviews, and those conducted with medical professionals by the author and for the ‘Born in Sheffield’ study, provide a valuable insight into the reasons for particular decisions and behaviours, and the impact of them. Whilst oral history has been criticised for issues of reliability, as Szreter and Fisher have noted, the ‘dialogue with the present’ inherently part of the interview is a productive route to studying the past.^7^ The questionnaire respondents provide further diversity; the interviews and questionnaire together ensure access to a relatively large number of parents’ experiences, and an in-depth perspective on individual experience, allowing for discussion and analysis of not only men’s presence or absence at birth, but its meaning to professionals and parents.^8^

This period saw substantial shifts in both medical and lay attitudes to and experiences of childbirth, with the decline in infant and maternal mortality in the first half of the twentieth century (the latter particularly dropping after a high in 1934), the introduction of the National Health Service in 1948, the growth in the numbers of women giving birth in medical institutions, the rise in patient choice and the challenge brought about by second-wave feminism to medical authorities, the increase in technologies available to manage pregnancy and birth, and the growing visibility of childbirth—and advice literature about it—in various media.^9^ As Beier argues, in parallel with other experiences of health and illness, the medicalization of childbirth was ‘inevitable and irresistible’.^10^ As Oakley and Tew have noted, the decrease in maternal and infant mortality across the century was as much, or if not more, due to the increased health of mothers rather than medical intervention.^11^ One of the most important developments in experiences of childbirth for mothers was the location of birth, as more women gave birth to at least their first child in hospital. The number of births in medical institutions rose in interwar Britain, from 23 per cent in 1923 to 35 per cent in 1937.^12^ The establishment of the NHS made an institutional birth with professional midwives accessible to more women. By 1954, 63.7 per cent of births took place at hospital, and this number increased rapidly between 1963 and 1972 when the rate of hospital births grew from 68.2 to 91.4 per cent.^13^ The changing location of birth raised questions about who should be present during labour and delivery.

A brief glance at the information available suggests that the change towards men’s presence during childbirth was a rapid one, shifting from a minority in the 1960s to a vast majority by the end of the 1970s. Locating precise figures is difficult, as hospitals did not tend to keep records, but government reports and guidelines tended to endorse this change. As McIntosh has argued, the Cranbrook and Peel Reports codified current practice, catching up with women’s wishes regarding the location of birth, rather than instigating change.^14^ In 1959, the Cranbrook Report recommended that 70 per cent of births should take place in hospitals, and added ‘In hospital her husband or a relation should be allowed to stay with her, if she wishes it, at least during the first stage of labour.’^15^ At a local level, the shift to allowing men into the delivery room happened in response to demands from parents and staff themselves, as well as a practical necessity of finding ways to support women. Groups like the National Childbirth Trust (NCT) and the Association for Improvements in the Maternity Services (AIMS) also pushed for this change, as part of wider campaigns to improve conditions for women in labour, along with significant individuals such as Sheila Kitzinger. In parents’ and practitioners’ accounts, there was little recognition of their specific influence. Yet, such bodies and schools of thought were important in creating a different atmosphere, arguing for choice and power for women. Feminist research challenged patriarchal conceptions and assumptions about childbirth.^16^ As McIntosh notes, AIMS and the NCT, as well as obstetricians and midwives, apparently in the face of opposition from each other, claimed they were responsible for the move to allow men’s presence.^17^

It seems that a major change towards men’s presence at childbirth happened over roughly a 10-year period, from the late 1960s to the 1970s. However, there are signs that the move towards greater involvement of husbands in childbirth could be found *before* the shift to hospitalisation, as discussed below, though assessing this statistically is difficult because of the limited availability of records. Some evidence of men’s involvement during labour and even delivery can be seen in the 1940s and 1950s, which was then challenged by the shift towards institutional childbirth. Husbands were, often literally, shut out due to strict institutional rules. When hospitals began to rethink their regulations around visitors and supporters for labouring women, the social changes that had originally encouraged a change in men’s role in home births led to a rapid shift towards their presence becoming the norm. Furthermore, other key shifts muddy the water of the idea of such rapid transformation: from men getting involved in some part of the process to being a more ‘active’ player in pregnancy, labour and delivery, and from fathers attending birth in certain situations to this becoming the norm across the country, in most birth scenarios and amongst all social groups. As Beail and McGuire noted by 1982 ‘The medical profession has now accepted that fathers have a right, if they wish, to see their children being born’. They added that fathers’ participation still seemed ‘limited, and few are allowed any form of active role’, although they suggested this was changing.^18^ And whilst men’s presence rapidly became normalised, this was something of a quiet revolution, indicating that men’s presence in the delivery room fitted with wider social norms. As Pat, a midwife who worked in hospitals in southeast England, noted, ‘I don’t think there was a sort of crash bang wallop and now fathers come in, I think they just sort of slithered in’.^19^ The discussion of this matter in *The Lancet* and *BMJ* was very limited, with few articles and letters tackling this subject directly.^20^

## 1940–1970

Few records of numbers of men attending birth at home or in hospital exist, but social surveys can give some insight into the levels of attendance. Newson and Newson, in their study of around 700 Nottingham families in the late 1950s, found men were present at the delivery in 13 per cent of home births. In contrast, no lay person, husband or otherwise, was present in a single case of hospital births.^21^ Similarly, a survey conducted by psychologists Woollett, White and Lyon in the early 1980s found that in 15 per cent of deliveries between 1940 and 1969, the father was present.^22^ Again, these were all home births; the location mattered in determining whether men were present. Pat Callis, a midwife from Sheffield, noted how novel and potentially frightening men’s involvement in birth was in the early 1960s, but suggested that ‘At home we used to get them a little bit more involved’.^23^ Whilst the numbers of men attending birth at this time were small, it is noteworthy that the limited evidence available suggests this was more likely at home than in hospital.

Being ‘present’ for a birth could mean a number of different things, including being there during the labour and/or the delivery itself. Crucially, when giving birth at home, the line between presence and absence was more blurred, as men were more likely to be nearby or in and out of the delivery room because it was a familiar space. Some midwives were at the forefront of this change. Harry and Rose had children in 1948 and 1952 in West Yorkshire, and Harry was present for the second labour and delivery, at home. They described how their newly-qualified midwife discussed the prospect of his presence, telling Rose ‘they’ve started now believing that fathers should be given the chance to be at the birth’. She added ‘now, some husbands only want what comes beforehand’. Harry didn’t want to be there at first, but when Rose explained that ‘most men’ wanted only the preamble to pregnancy, he thought ‘that makes me sound awful’. Harry had imagined ‘peering in’ and ‘a gory scene’, but he sat next to Rose, and held her hand.^24^ Harry and Rose were sure they were very unusual, and were one of the first couples in the West Yorkshire area to do this. Another interviewee, Harold, was not allowed to be with his wife during her first labour, in 1962, in hospital. Although, tragically, it was a stillbirth rather than twins as they had expected, he remembered ‘they wouldn’t let me sit with her or anything like that’. In contrast, two years later, they had a son at home, and Harold said ‘I were there when he were born and I held him straight away’ [ … it was] ‘a lovely morning’.^25^

In contrast, ‘presence’ and ‘absence’ were more formally defined and regulated in hospital. Initially, some hospitals were more welcoming than others: University College Hospital London invited fathers into their delivery rooms as early as 1951. As an obstetrician explained to a BBC interviewer in 1953,

we do encourage fathers—if they want to come. Er, sometimes they feel they don’t want to be present, nor do their wives want them to come, but we’ve had quite a number now of expectant fathers who’ve been present at the time of delivery. Most of them stand up to it very well. … On the whole it’s a terrific and exciting experience for both expectant father and mother.^26^

Other practitioners were strongly against fathers’ involvement. Dr Patterson of Tyrone County Hospital wrote in the *British Medical Journal* in 1961,

Let us not pander to morbid curiosity and sensationalism, nor to those featherbrains who wish to be in the van of a new fashion, by encouraging a highly unnatural trend with the mumbo-jumbo of pseudo psychology. The proper place for the father, if not at work, is the ‘local,’ whither instinct will usually guide him. Family men may be baby-sitting, unless ejected by mother-in-law.^27^

Indeed, hospitals outside of London often took longer to allow fathers in. Mary recalled asking whether her husband Alf could stay with her for their first child’s birth in hospital in Leeds in 1961: ‘I said I don’t know why husbands aren’t allowed to be here’, and asked ‘what would happen if I had a screaming fit until you let him in’. The nurse replied ‘you’re not going to have a screaming fit’. Alf was not present for the labour or delivery.^28^ Sheffield’s Jessop Hospital changed its regulations in 1968.^29^ According to midwife Mary Croft, pressure from midwives on consultants instigated the change. She described telling a senior doctor that midwives wanted men to stay with their wives. His initial response was ‘rubbish, rubbish’, but she persisted, highlighting that ‘it’s 1968 … and we have to move with the times’.^30^ The success of these midwives suggested that the hospital had not kept pace with wider social changes, although not all midwives completely agreed with the idea. Barbara Ford said ‘It seemed strange. To a single person who had never been married and not being worldly-wise I thought it was awful that men were there.’^31^ Visiting hours could also prohibit men’s involvement after the birth in ways that might be possible at home; Henry recalled being unable to see his new baby until the following day, after her birth in a Lincoln hospital in 1965. She arrived around 10pm, ‘so of course I couldn’t see her then’. He was present for the births of his other three children, at home in 1968 and 1970 and in hospital in 1975.^32^ In other cases, husbands were only admitted to the labour and delivery in certain circumstances; Mike recalled when his first child was born in 1971 in Middlesbrough, the matrons were very much in charge, so ‘it had to be special circumstances’ if men were present.^33^

The reason for men’s absence during the birth of their child, where it was articulated at all, was often framed in gendered terms. In the immediate post-war era, men, women and midwives usually defined their opposition to men’s presence at childbirth on this basis: men were not welcome because they were men, and men didn’t want to be there because it wasn’t manly.^34^ Glyn, whose first child was born in hospital in 1948, stated that he phoned the maternity ward every five minutes to check on progress, but said: ‘Oh there was no suggestion at all of your being with your wife at my time’.^35^ Fred, who became a father in 1961, noted ‘It wasn’t a man’s province in those days’, and added he wouldn’t have wanted to be there. The delivery room was simply not a ‘man’s place’, even if men were keen to be supportive during labour and see their new baby just after it was born.^36^ Women frequently agreed: Mary, a mother of five born between 1954 and 1964, was horrified by the prospect:

 Interviewer:  Was Ronnie there?

Subject:   What—that was disgusting.

Interviewer:  That wasn’t seen to be … ?

Subject:   Oh my God, no way, nobody mentioned that in them days. For a man to see you like in that state. Eeh no.^37^

Ideas of privacy were highly gendered; who should and should not see a woman giving birth was dictated by their gender more than any other variable, although medical qualification could override this norm, as male doctors attended women. Only a minority reflected that they wished they or their husbands could have been present, but this had not been possible or allowed.^38^ By the late 1960s and into the 1970s, expectations were changing; Ben was told he was welcome to attend his first child’s birth in a Reading hospital in 1970. However, when his wife was taken away for a forceps delivery, he recalled it was clear ‘there was no way I could go in’ and reflected ‘I was quite disappointed I must admit that I didn’t actually see [my daughter] born’.^39^

Men were often around whilst their wives were labouring, and were happy and eager to see their child soon after it was born, something which was usually more possible at home. Brian noted that whilst he was there for a large part of the birth, when it came to the delivery he remembered that his wife’s mother ‘said you can get out, she said, this is nothing to do with men’, again reflecting the strongly gendered assumptions around childbirth. As husbands and fathers, men were increasingly expected to ‘help’ and support their wives emotionally.^40^ Yet, the physically intimate tasks of childcare remained women’s responsibility, and a separation from childbirth and infant-care remained important to constructions of masculinity in this period, even as norms around fatherhood were shifting substantially.^41^ Yet, it was often a hugely significant moment for fathers: men who did not want to witness the delivery itself also described the strong emotions they felt. John described how he was ‘proud as punch’, though he was keen to stress he did not cry: ‘I just filled up. I didn’t properly cry or that, I just filled up you know, with emotion’.^42^ Glyn said that he was ‘Proud, extremely proud. I wanted to tell everybody, you know’.^43^ Henry, though not involved in the birth, recalled how much he felt his life and he, himself changed in the wake of becoming a father in 1965.^45^ Strong gendered norms and institutional rules could therefore militate against men’s involvement in the birth process or presence at delivery. However, small changes were taking place with some men becoming more involved in births at home. Numerous men sought ways to feel involved and considered this a moment of significant change in their life course, from the retrospective moment of the interview, however involved they had been in the birth itself.

## 1970s–1980s

Throughout the 1970s, the numbers of men attending birth increased. In Woollett *et al*.’s study, fathers were present at 71 per cent of deliveries in the 1970s, most of which were in hospital, and a number of studies found similar results across Britain in the late 1960s and 1970s.^44^ By the 1980s, this reached new levels: Bell, McKee and Priestley, in their 1983 study, found that 38 men in their sample, or 13.5 per cent, did not attend any part of the birth of their child.^46^ In Jackson’s study published in 1984, 82 of 100 fathers were present, and he highlighted the speed of this change: ‘From zero to over 80 per cent, within a decade, is evidence, repeated elsewhere, of a shift in the expression of male feeling and male commitment’, although his assertion of ‘zero’ men attending birth before 1970 is questionable, as we have seen.^47^ Finally, Lewis found 85 per cent of men attended some part of the labour in the mid-1980s, with 67 per cent attending the delivery.^48^ Whilst Lewis made this distinction, some researchers did not. As discussed above, men could be at least nearby during home births, and some, like Alf, were present right up until the baby’s head emerged. But rules and regulations dictated men’s involvement in the labour and delivery wards in hospitals and maternity homes, and such rules could up until this point eliminate all possibility of men’s presence.

**Fig. 1.**
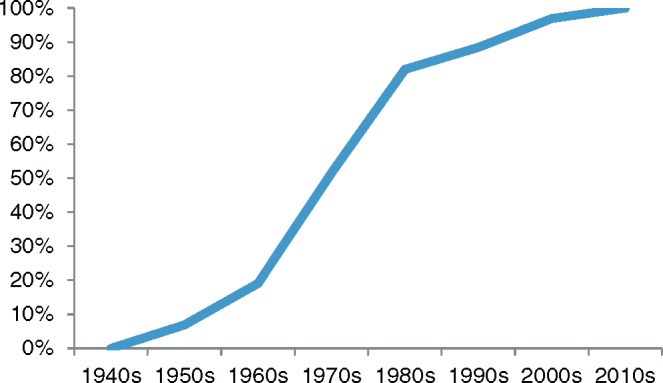
Percentage of men attending delivery. Total number of births: 310.

Figure 1 illustrates the numbers of men present in personal accounts from original and archived interviews and the online questionnaire, totalling 310 births. In analysing this material, each birth is recorded separately and whether the father was present at the moment of delivery or not: the data refer to individual men’s experiences of multiple births. These data are a partial picture; however, this graph demonstrates the increasing likelihood of presence in the 1970s.

The increase illustrated in Figure 1 is largely in accordance with social research, indicating the 1970s as a decade of rapid change. From the individual accounts, between 1970 and 1974, men were present for 22 of 57 deliveries (39 per cent) and between 1975 and 1979, 29 of 42 births (69 per cent). Indeed, although the number of births in each year is small, there was a growing probability of presence as the 1970s progressed.^49^ Furthermore, in the 1970s, in most of the deliveries for which men were not present, they had been with their partners throughout and often intended to stay, but a complicated delivery meant they chose or were asked to leave for the final minutes. From 1975, male partners were absent for a reason other than a complicated delivery in only 16 of 201 deliveries—and in some cases, the couple wanted him to be there but circumstances prevented it.^50^ Collectively, social research and individual accounts demonstrate that a minority of fathers attended delivery up until the late 1970s and 1980s. After an initial and gradual shift towards men’s presence in home births in the 1950s and 1960s, and a curtailing of this change by the move to hospital births, from the late 1970s men’s presence at delivery in hospital seemed to have become the norm.

Some hospitals had specific policies determining when men could be involved in the labour and delivery. A number of hospitals and medical practitioners were happy to facilitate men’s involvement during labour but were uncomfortable with the idea of men witnessing the apparently more undignified second stage, something which had also been common in home births previously. Meryl Thomas described how change occurred in St David’s hospital in Cardiff and in the Royal Berkshire hospital in Reading in the 1970s. In Cardiff, she described how ‘we then erm started to have fathers in the labour ward, erm and they would be with their wife all through labour and then we would ask them to step outside while she was in the second stage’.^51^ She moved from Cardiff to Reading in 1974, and contrasted the two hospitals: Reading was more influenced by contemporary ideas, most notably those of obstetrician Michel Odent. When she moved, staff were starting to encourage men’s presence during the whole birth. She summed the change up as, ‘[in the] early 70s fathers started to be there, but not for the delivery, by the mid-70s they were there for the whole thing, by the 80s they were [pause] part of it’, and highlighted the impact of NCT thinking on parents’ wishes.^52^ Cathy Warwick, current General Secretary of the Royal College of Midwives, agreed, highlighting that during her training in the mid-1970s, she believed fathers were around for labour and delivery. Her first recollection when asked about men’s involvement was a father who read his newspaper throughout. She suggested that whilst some women included their partners and others in the birth, she thought ‘the guy with the newspaper was probably more typical at that time, er, the guy who was there but not particularly involved’.^53^

However, Cathy Warwick remembered her early experiences of community midwifery attending to women in their homes, in which she described how ‘of course all of those fathers were very involved [Mmm] erm, virtually in the same way as they are now’.^54^ Liz Stephens agreed and spoke in detail about the various advantages of home birth or birth in small midwife-led centres.^55^ Mavis Kirkham, a midwife from Sheffield and later researcher, similarly suggested the place of birth was the most important variable in determining male partners’ involvement.^56^ All three trained in the 1970s, and suggested that even when women were increasingly giving birth in hospital, and hospitals were changing their policies to facilitate fathers’ involvement, men were more likely to be not only present but involved during labour and deliveries at home, through physical and emotional support for their labouring partners.

The presence of men in the labour and/or delivery room had consequences for medical staff. Some practitioners highlighted potentially positive effects for midwives and women. Tom Smith, a doctor in Sheffield, thought in retrospect that the involvement of husbands was a wholly ‘good thing’, as it meant childbirth was no longer a ‘very lonely business’.^57^ Others thought it helped them to help women, and made their jobs easier.^58^ Some staff spoke about fathers’ presence in terms of their authority. Myra Snell, though initially against fathers’ presence, realised it protected staff; the partner was an additional witness so ‘she couldn’t come out of the delivery room and say, ooh they did this, they did that they did the other [yes] because the dads were there!’^59^ This, she thought, could influence the couple’s relationship. However, medical practitioners could fear the questioning of their authority that another presence could bring about. Sheila Duncan, a senior doctor and lecturer in Sheffield was pessimistic, noting ‘If the husbands had read all the books and decided how it was going to run they could be very assertive in some circumstances.’^60^

Men often expected to be led by medical staff, their wives/partners and other female family members.^61^ Edward and Dorothy described the expectations of fathers in the mid-1970s, noting ‘they certainly weren’t welcome at antenatal classes or to go in the hospital to look round beforehand that just wasn’t [pause] considered like it is these days’.^62^ Yet, it was clear that some couples were able to carve out a role for fathers. Martin, speaking about his second child’s birth in 1970, noted ‘my job was just to hold her hand, and try to calm her down while the nurses did the job’.^63^ Likewise, Michael, who had two children in 1972 and 1974, focused on his role as a partner, highlighting that ‘I didn’t watch it or nothing like that. I was more concerned with [my wife], you know. Sorta keeping her calm and things.’^64^ One or two fathers highlighted how it brought them closer to their wives; Gerald was there at both his children’s births in 1978 and 1980, and noted that ‘I mean it brings you really close together, because I think the woman has to go through a helluva lot. All sort of misshapen and everything for all that time, and I mean the father doesn’t do that much. I think for him to be there—it does help em appreciate and really bonds you together.’^65^

For whatever reason they chose to be there, most men focused on supporting their wives *and* witnessing their children entering the world. A questionnaire respondent writing about his children’s births in 1967, 1968 and 1980, described them as ‘the most memorable and moving moments of my life’, a very common theme.^66^ Peter, describing his second child’s birth in 1973, noted that ‘It’s such a wonderful thing to see’ and regretted that he wasn’t allowed to witness his first child being born.^67^ Fathers who had mixed feelings about becoming a parent spoke about the emotional significance of the birth; Robert, who became a father in 1978, highlighted

I think that, I think if I hadn’t have been at the birth, there might have been a, like I might have been able to split away and not see her, but ’cos I was at that birth I think that, I mean I’d go again tomorrow to see it. It was terrific like. I mean I don’t think I would never have seen her, but after we split up I think it made me want to see her more.^68^

Yet some men felt underwhelmed or isolated by the experience. Men who had been present in the 1970s sometimes resisted the emotional expectations placed on childbirth in the cultural context of interview in the 2010s. Edward found his only child’s birth in 1976 fine but not as awe-inspiring as others claimed: ‘I can’t really ever say that I was delighted and over the moon that I’d got a son’—‘that’s not me’.^69^ Barry recalled that after his son’s birth in 1972, he was ‘pleased, but I wasn’t ecstatic. Er, just the sort of next stage to go through’.^70^ Others highlighted how traumatic or distressing birth could be. Leslie Lane was planning to be at his daughter’s birth in 1969, but left as the baby’s head was emerging. He explained, ‘you can watch anything on the television—if it’s someone else, it’s remote, but when it’s someone you know, then it’s a bit different’ and summarised the experience as ‘thrilling, but also frightening’.^71^ None of these men saw being present for the birth as radical, highlighting the rapidity with which this became ‘normal’ for the generation becoming parents in the late 1970s and early 1980s as compared to their parents. Despite the growing normalisation of men’s presence at birth in hospitals, it is clear that medical regulations and the attitudes of medical practitioners remained influential in determining men’s participation in childbirth.

## 1980s to the Present

Since the 1980s, the attendance of men at their children’s births has become almost completely normalised. In 1985, in Niven’s study of 98 fathers, 79 were present for most of the labour and delivery.^72^ In 1992, a study comparing Greece and Britain found that 91 per cent of the British sample of 142 fathers were present, and in contrast to Greece, ‘attendance at delivery is the norm and social pressures deter non-attendance’.^73^ By 2000, in Singh and Newburn’s study for the National Childbirth Trust, 96 per cent of their sample of 463 men attended some part of the birth, with 88 per cent attending the delivery itself. They found no major differences across class, age and ethnic groups.^74^ They differentiated between different types of birth—men were present at the point of delivery in 96 per cent of vaginal births, 96 per cent of assisted births, 77 per cent of emergency Caesarean sections (C-sections), and 84 per cent of planned C-sections. In a study of babies born between 2000 and 2001 in Britain, by Jayaweera *et al.*, the father was present in 86 per cent of cases overall, 93 per cent of cases when the mother and father were together, and 47 per cent of cases when the parents had separated.^75^ This study, in contrast to that of Newburn and Singh, highlighted a lower rate of fathers’ attendance amongst ethnic minority groups. The variance in behaviours across groups from different ethnic and religious backgrounds was noted by some of the interviewed midwives; Pat noted that Orthodox Jewish men were potentially less able to support their partners during labour as religious beliefs prevented them touching a bleeding woman, whilst Sandra and Sheena Byrom highlighted how South Asian men were in their experience less likely to participate in the birth process until much more recently.^76^ The differences between ethnic and religious groups warrants further investigation.

Throughout this period, midwives and doctors continued to play a powerful role in determining how men participated in the labour and delivery. Mavis Kirkham described one consultant’s obstinacy regarding C-section births. Whilst men’s presence and involvement in ‘normal’ births was standard by the 1980s, their presence in instrumental births was not as widely accepted, and the involvement of partners in C-section births came later.^77^ C-section births increased in prevalence in the late 1980s and 1990s, reaching 15.5 per cent in 1994 and 22 per cent in 2001/2.^78^ In Nether Edge hospital in the early 1990s, a senior consultant completely refused to allow husbands to attend; no amount of campaigning from midwives or parents had any impact. Mavis spoke about how she helped parents ask him repeatedly to change his mind, down to how ‘it was really important to wear a suit and look respectable and call him Sir’ when they did so, demonstrating the greater likelihood of those with a middle-class demeanour being able to effect change. Change finally happened when he went on holiday for some weeks: she explained ‘his locum was a woman obstetrician … anyway you know this couple donned their respectable gear and booked an appointment and erm asked this lady and she said of course now you’ll need to be outside the theatre at this sort of time’. Mavis described how change was consolidated:

this was slightly orchestrated, when the er eminent gentleman came back from his holiday he had a stack of thank you letters on his desk erm, all from couples whose lives were you know made infinitely better as parents by the presence of the husband in his theatre and how enlightened his firm was and erm he crumbled, and you know, that was it. But it was orchestrated and it wasn’t because anybody who was routinely doing the surgery in that unit thought it was a good idea, they were shamed into it.^79^

This is a clear example of parents and midwives rather than senior staff or policies driving change. Mavis remembered this as a period in which generally women were pushing for change, not only around partners’ presence but also for alternative birth positions and other individualised practices, both within hospitals and in political demonstrations.^80^

The changing nature of midwifery care could also enable men’s more ‘active’ involvement in birth, where by they were not just physically present, but were an active participant, through physical and emotional support for their partner and immediate bonding with the baby. Cathy Warwick noted that fathers at the time of interview, in 2012, could have skin-to-skin contact with their baby, or in some hospitals stay overnight after the baby was born. Yet, she reflected that midwives in the 1970s and 1980s were ‘busy cutting cords, and clamping cords, and getting the baby dried, and not that you don’t do that now, but sort of sorting the baby out as opposed to ensuring a positive relationship, so it would have been quite hard for fathers to behave differently, because we, the whole context was different.’^81^

Once fathers’ involvement became more usual, however, midwives also noted that fathers could be over-zealous, to the detriment of their partner’s care. Cathy Warwick described how in the 1980s, one woman wanted analgesia, which her partner opposed. She recalled ‘I remember thinking … he’s, it’s almost like it’s his birth as opposed to her birth, and his preferences are dictating—are—sort of, he’s wanting them to influence her’.^82^ Indeed, Meryl Thomas recalled a case in which one man, highly qualified in his profession, told her that his partner was ‘not getting on very well, so I’ve increased it’, referring to the dose of syntocinon Meryl administered to speed up the labour.^83^ Liz Stephens thought gender dynamics were very important, noting ‘it comes back into the patriarchal, whole patriarchal emphasis in maternity services, it comes back to “we’re men and we know what’s best for you”’.^84^

Men were unlikely to describe themselves in such terms in an oral history interview context. However, Ali, who became a father in 2004, described how he had planned to be the first to touch the baby, and when the baby’s head emerged, the ‘consultant was next to me and I just gently nudged her out of the way and carried my child as she came out’. This was something he enjoyed, had planned out, but had not discussed with his wife. He also asked that no men should be admitted into the labour room, which he felt was not well respected by the medical staff. When his wife was pregnant for the second time, Ali described telling her to stop taking prescribed medication ‘because at that particular point you need to get your head and your body and everything else, really thinking right’. This led to ‘conflicts’ between the couple and with medical professionals, and Ali noted ‘my wife would tell you I wasn’t probably the best, the best husband at that particular point’.^85^ Whilst parents appreciated the choices afforded to them as the period progressed, this brought about a greater willingness of some men, particularly those who felt enabled by their social position or religious or political beliefs, to challenge the authority of medical staff and even their partners’ wishes. In an American context, Leavitt suggests that this development reinforced traditional authority structures, of the medical profession and of men within families.^86^ However, Bryder, focusing on New Zealand, found little evidence of this, suggesting instead that men remained marginalised and often powerless figures.^87^ Accounts from medical professionals and men themselves demonstrate this to be largely true of the British context. Despite accounts of a small minority of men taking an overly directive role, a much larger number of men pointed to their feelings of helplessness and unease.

Indeed, a significant minority of fathers found the experience terrifying or, because the birth involved various complications, they did not enjoy it. This theme was much more common in men’s accounts of births from the 1990s onwards, as the pressure to be there and be a helpful, supportive presence grew. As in Brown’s 1982 study, men could feel helpless, an encumbrance or redundant if the birth was highly medicalised.^88^ The interview evidence largely mirrors Brown’s findings; there were three ways in which men found their experience of birth to be difficult: some men felt largely useless, even if their partners valued their presence; some men felt inextricably isolated; and others found it distressing, particularly if things didn’t progress smoothly. Gary enjoyed the birth of his first son in 1997 and thought it could be an important bonding process, but felt pressure to take part in certain aspects of it. He explained ‘I’ve been to forced to cut the umbilical cord on both occasions and I didn’t want to’. He noted a high degree of pressure to be there, but ‘maybe it’s not right for everybody, maybe you don’t want to be there’.^89^ Matt, whose first child was born in 2013, said that ‘brutal is the only word I can use to describe childbirth’. He had done some research and thought he was prepared, but added ‘I was sadly mistaken!’ He also described ‘People say to you ahhh, when your baby’s born and you hold it, you know, you can’t explain what that feels like, it’s just incredible, and you can’t—for me it wasn’t. … It wasn’t love or elation, but just awe I guess’.^90^

Men’s recollections of uselessness were one of the most dominant themes across the interviews. Many highlighted that they ‘felt out of my depth’, ‘unhelpful’ (James, first child born 1986); ‘useless’, ‘you can’t do anything’ (Jeremy, first child born 1991); ‘a bit like a spare part, at times’, (Steve, first child born 1994), ‘a loose end’ (Gary, first child born 1997), a ‘bystander in a way’ (Charlie, first child born 2005), and ‘you’re an appendage to the main event’ and a ‘bit part player’ (Carl, first child born 2006).^91^ This particularly affected Andrew, who became a father in 2009 and said he was ‘worried and stressed’ leading up to the birth. He felt he ‘had to at least pretend to be confident’, but was very anxious.^92^ Similarly, Bill described the experience of his first daughter’s birth in 1992, as ‘tough’ from a man’s point of view, because ‘I didn’t know if I’d be able to be that strong person for her’.^93^ This suggests that the increased pressure for men to be an important part of the process actually left them feeling stressed and not supporting their partners as well as they might. Aidan’s experience reflects the growing pressures and expectations on both parents, to enjoy the birth and feel immediately connected as a family. Aidan had two children, the first born in 2002. He simply assumed he would be involved in everything, but felt largely redundant, ‘semi-detached’ in Aidan’s words, as his role was simply ‘cheering from the side-lines’. He added ‘you’re a bit part really, you try your best to be supportive but what can you do really’.^94^

Most men, despite such feelings, were pleased they had witnessed their children’s births. As men’s involvement in childbirth became commonplace, the emotional and social significance of ‘being there’ increased. Some parents highlighted that the father’s presence was about future bonding between parent and child, particularly those who had children from the 1980s onwards.^95^ This was a notable theme in Kirkham’s observational research in the 1980s.^96^ Indeed, the emphasis on the importance of welcoming one’s child into the world as a father has been increasingly acknowledged since the 1980s; this is part of the rationale for men’s presence and influences how medical staff treat men.^97^ Indeed, the expectation that fathers not only could but should be present for their child’s birth also increased the sense of disappointment if men were absent. Dean, who first became a father in 1998, described how he missed the birth of his first child because he was incarcerated during his girlfriend’s pregnancy. He felt he’d ‘abandoned my child, felt I was replicating what my father did’. However, he linked becoming a parent to important changes in his life regardless. Dean recalled starting to think about ‘a bigger picture’, noting he wanted ‘to be there for starting nursery, school, getting her through the system I didn’t’.^98^

The context to these developments in men’s involvement in childbirth was the changing balance of authority between medical professionals and parents and the medicalisation of childbirth, and concerns about this underlay much debate about men’s involvement. Many midwives interviewed talked negatively about the institutional rules that turned the birthing mother and her family into patients—some highlighted the somewhat institutionalising approach that could be found in most hospitals, particularly in the 1970s and 1980s. Women could be stripped of their individuality and institutionalised through a compulsory bath, pubic shave, enema and hospital gown. Midwives likened units to ‘conveyor belts’ in the late 1970s and 1980s, and Liz Stephens, former President of the Royal College of Midwives, suggested that maternity units in hospitals still resemble ‘Fordist production lines’.^99^ Men too were subject to this sort of approach: medical staff and institutional rules could isolate and disempower them.^100^ As Kirkham highlighted, men had no role or status, and the most frequent request made of them in her 1980s study was, simply, to leave.^101^ Yet a more natural approach, encouraged by bodies like the NCT and particular groups of midwives, helped create an alternative understanding of childbirth as a family, even social or somewhat public, event—‘a normal physiological family life event’ as Liz Stephens suggested. As such, she argued for personalised birth centres in which the family were given their own space, in contrast to the ‘alien environment’ of the hospital delivery suite.^102^ Many midwives, furthermore, pointed to the new emphasis on patient choice as the most dramatic change in maternity services over recent decades, and this included whether and how to involve partners.^103^ The influence of the natural childbirth movement and the increasing involvement of fathers were arguably mutually reinforcing trends across this period.

## Conclusion

This article argues that attitudes and practices around men’s involvement in childbirth were starting to change in the 1950s. Most couples did not consider men’s presence to be possible or desirable, but a small minority, recognising they were out of line with their peers, such as Harry and Rose quoted above, embraced this idea. Their accounts, highlighting their difference from previous generations and their peers, and suggesting they were at the forefront of a longer-term change, consolidates what the limited statistical evidence can tell us: that this change in men’s involvement took place on a small scale at home, a shift that was then discouraged by the hospitalisation of birth. Hospital rules also limited the ways in which men had been involved in their children’s births previously, such as in the early stages of their wives’ labours, or in helping out immediately after the baby was born.^104^ As institutional rules changed over subsequent decades, from the early adopters of this practice such as University College Hospital to the inclusion of men in C-section births in the 1990s in Sheffield, the idea of men’s involvement became rapidly normalised. As the proportion of men attending shifted from a minority to a majority in the 1970s, couples no longer thought this was an unusual or radical act, and often simply assumed the father would be present. Yet, as the proportion of men attending has increased, their accounts also hint at a growing pressure to be a supportive and helpful presence, and disappointment when this was not possible or when the emotional experience of birth did not live up to expectations.

The reasons for this shift are multiple and complex. As Blackshaw notes, the shift to men’s presence during birth mirrored ‘the social changes that were occurring in work patterns and traditional social roles and identities’, as ideas about gendered identities were challenged in the wake of second-wave feminism.^105^ The views of key figures such as Grantly Dick Read, Kitzinger and Odent shaped the ideas of practitioners; as Bryder demonstrates, psychological thinking was significant.^106^ Institutional rules could certainly bring about change. But the evidence presented here demonstrates the importance of wider social changes as the root cause of this shift. If men’s involvement was starting to change at home first, before change appeared in hospitals, it was not the institution that drove change. Indeed much change at a policy or hospital level appeared to be reactive, as with the national Cranbrook and Peel Reports, as McIntosh describes, or the push for hospitals to ‘move with the times’.^107^

Furthermore, many interviews noted wider gendered norms when describing whether men were there or not for childbirth; whilst interviewees who had children in the early part of this period thought it was inappropriate for men to be present, by the end of the period, changing notions of masculinity meant that being around was desirable. One Scottish father, who became a dad in 2009, described ‘In terms of masculitity (sic) I felt that being there for the birth was a pretty manly thing to do. Being at the pub or elsewhere would have seemed really lame, and in my estimation not at all “manly”.’^108^ Indeed, medical practitioners are as much part of a changing cultural context as the women they care for, and it was the changing social backdrop of gender and family life that was influential in bringing about this shift. The move away from conceptions of birth as predominantly a medical phenomenon cemented the role of fathers in childbirth, as not just present but involved in physically supporting their partners and welcoming their child into the world. As Collier and Sheldon note, ‘If health care professionals aim to provide the best possible experience of a milestone in one’s life, then the needs of the father become far more important than if their exclusive goal is a medically safe delivery.^109^ As Bryder found in New Zealand, the involvement of men in hospital births contributed to and was part of an alternative understanding of childbirth as a social as well as a medical event.^110^ This changing context was in part encouraged by NCT and AIMS campaigns to improve conditions and choices for women giving birth in hospital.^111^ Whilst it was initially—in the words of Pat—‘middle-class trendies’ fresh out of NCT courses who were more likely to get as involved as possible in the birth process and challenge authority in order to do so, a wide range of men from a variety of backgrounds were present for their child’s birth, and enjoyed it.^112^ Furthermore, as Kirkham highlighted, the circumstances of the birth experience, such as the location and the level of medical intervention needed as well as the attitude of the midwife, often determined the nature of men’s involvement, rather than their background.^113^ Midwives also pointed out that the reactions of men were highly individual.^114^ For most couples, the change was a positive one, but this is not a simple story of progress. Some midwives, and even men themselves, discussed how men’s presence could have a negative impact on the birth experience for the woman involved: men could try to take control of the birth in a way that was potentially harmful. For couples suffering difficulties in their relationship, the man’s presence could inhibit the progress of labour.^115^ And the rapid shift towards this becoming very much the norm means that couples are less likely to think through who is best placed to support a woman through labour, whilst newer norms of masculinity reinforce the need for men to both be there and be strong. Men do not always live up to this standard. The discussion about the desirability of men’s presence continues.^116^

## Footnotes

1 Pat Jalland, *Women, Marriage and Politics, 1860–1914* (Oxford: Oxford University Press, 1988), 145; John Tosh, *A Man's Place: Masculinity and the Middle-Class Home in Victorian England* (New Haven, CT, and London: Yale University Press, 1999), 81–2.

2 Laura King, *Family Men: Fatherhood and Masculinity in Britain, 1914–60* (Oxford: Oxford University Press, 2015), 175–7.

3 Walter Greenwood [1933], *Love on the Dole* (London: Jonathan Cape, 1966), 238.

4
*Love on the Dole*, directed/produced by John Baxter (British National Films, 1941).

5 ‘Intimate Union’, BBC Television programme, broadcast 30 September 1964, <http://www.bbc.co.uk/archive/marriage/10508.shtml>, accessed 29 January 2015.

6 Original interviews by the author with midwives include a full name or pseudonym and date (e.g. ‘Interview with Pat (2012)’); archived interviews with midwives and doctors also include information about the location of transcripts. Eight interviews with midwives were conducted by the author in 2012. Interviewees were recruited through word of mouth and through the Royal College of Midwives’ networks. These, along with the archived interviews in the ‘Born in Sheffield’ collection as well as published interview collections, provide a snapshot of medical perspectives. Interviews were semi-structured and typically lasted from 30 minutes to two hours. Interviewees were given information and a consent form in advance before deciding whether to take part, and the chance to ask questions before, during and after the interview to ensure informed consent. Participants were able to make any restrictions on the use or storage of their interviews. Where requested, a pseudonym is used; otherwise, the midwife’s full name is given. Original interviews by the author with parents include a pseudonym and date (e.g. ‘Interview with Bill (2014)’); archived interviews with information about the location of transcripts. Fifty original interviews were conducted by the author between September 2013 and September 2014, with men currently living in the Yorkshire region, but originally from all over the UK (in seven cases, their partners also took part). These interviewees were recruited through a wide range of methods, including a radio appeal, contact with local community groups, advertising in local libraries and snowballing. Interviews were conducted with the same conditions around informed consent and format as above. All interviewees were assigned a pseudonym following the interview. Archival research into childbirth includes use of the Elizabeth Roberts 1940–70 collection (University of Lancaster); the ‘100 Families’ collection conducted by Harold Newby and Paul Thompson available online through the UK Data Archive; and Steve Humphries’ two collections, ‘A Man’s World’ (at the British Film Institute Archive), and ‘A Labour of Love’ (at the British Library). An online questionnaire for parents was hosted on the University of Warwick website, asking parents to answer a short survey about their experiences of childbirth. Respondents were recruited by advertising on social media and through parents’ groups. Participants were asked whether the father of their child was present for each birth and an open question about their experiences of labour and delivery. Respondents were asked for their consent for their information to be used in research, and given information about the project, as well as contact information to find out more. A total of 117 parents based in the UK responded.

7 Simon Szreter and Kate Fisher, *Sex before the Sexual Revolution: Intimate Life in England 1918–1963* (Cambridge: Cambridge University Press, 2010), 11–13.

8 For more on using interviews in historical research, see April Gallwey, ‘The Rewards of Using Archived Oral Histories in Research: The Case of the Millennium Memory Bank’, *Oral History*, 2013, 41, 37–50. On oral history methodologies and uses, see Alessandro Portelli, ‘What Makes Oral History Different’, in Robert Perks and Alistair Thomson, eds, *The Oral History Reader* (London: Routledge, 2006), 32–42; Penny Summerfield, *Reconstructing Women's Wartime Lives* (Manchester: Manchester University Press, 1998), 1–42.

9 On the decline in infant mortality, see Nicky Leap and Billie Hunter, *The Midwife: an Oral History from Handywoman to Professional Midwife* (London: Scarlet Press, 1993), 146–7; Tania McIntosh, *A Social History of Maternity and Childbirth* (Abingdon: Routledge, 2012), 46–54. On the introduction of the NHS, see Angela Davis, ‘A Revolution in Maternity Care? Women and the Maternity Services, Oxfordshire c.1948–1974’, *Social History of Medicine*, 2011, 24, esp. 401–2; Leap and Hunter, *The Midwife*, 16–17; McIntosh, *A Social History*, 73–6. On the move from home births to in-patient deliveries, see Julia Allinson, *Delivered at Home* (London: Chapman and Hall, 1996), esp. 41–59; Lucinda McCray Beier, ‘Expertise and Control: Childbearing in Three Twentieth-Century Working-Class Lancashire Communities’, *Bulletin of the History of Medicine*, 2004, 78, 404–8; McIntosh, *A Social History*, 63–7, 107–13; Ann Oakley, *From Here to Maternity: Becoming a Mother* (Harmondsworth: Pengiun, 1986), 15–16; Geoffrey Rivett, *From Cradle to Grave: Fifty Years of the NHS* (London: King’s Fund, 1997), 71–2, 153–4, 226–7, 403. On challenges to medical authority, see McIntosh, *A Social History*, 113–28, 131–4 and on the increasing use of technology, *ibid.*, 101–7. The first birth shown on television was in 1957, for example. Sarah Clement, ‘Childbirth on Television’, *British Journal of Midwifery*, 1997, 5, 37–42.

10 Beier, ‘Expertise’, 380.

11 Oakley, *From Here*, 15; Marjorie Tew, *Safer Childbirth? A Critical History of Maternity Care* (London: Chapman and Hall, 1995), xii.

12 McIntosh, *A Social History*, 64.

13 Davis, ‘A Revolution’, 390; Ann Oakley, *The Captured Womb: A History of the Medical Care of Pregnant Women* (Oxford: Basil Blackwell, 1984), 215.

14 Likewise, the Peel Report of 1970 with its encouragement of high rates of hospital birth, reflected change that was already taking place. McIntosh, *A Social History*, 90, 110–11.

15 Ministry of Health, *Report of the Maternity Services Committee* (London: HMSO, 1959), 16.

16 See Oakley for a discussion, and example, of this. Oakley, *From Here*, 22–4.

17 McIntosh, *A Social History*, 119. On the American context, see Judith Walzer Leavitt, *Make Room for Daddy: The Journey from Waiting Room to Birthing Room* (Chapel Hill: University of North Carolina Press, 2009), esp. 287.

18 Nigel Beail and Jacqueline McGuire, ‘Fathers, the Family and Society: The Tide of Change’, in Beail and McGuire, eds, *Fathers: Psychological Perspectives* (London: Junction Books, 1982), 241.

19 Interview with Pat (2012).

20 The few examples include ‘Fathers at Delivery’, *British Medical Journal*, 1961, 1:5225, 594–5; T. N. A. Jeffcoate, ‘Review of Robert A. Bradley, *Husband-Coached Childbirth*’, *British Medical Journal*, 1966, 1:5483, 350–1; ‘Father in the Labour Ward’, *The Lancet*, 26 March 1966, 699; Sidney Crown, ‘Father in the Labour Ward’, *The Lancet*, 9 April 1966, 815; B. H. Burne, ‘Father in the Labour Ward’, *The Lancet*, 7 May 1966, 1038.

21 John and Elizabeth Newson, *Infant Care in an Urban Community* (London: George Allen and Unwin, 1963), 28–9.

22 Anne Woollett, David White and Louise Lyon, ‘Observations of Fathers at Birth’, in Beail and McGuire, eds, *Fathers*, 73.

23 Interview with Pat Callis, ‘Born in Sheffield’ collection, Sheffield Archives, 14.

24 Interview with Harry (2013).

25 Interview with Harold (2013).

26 BBC, Brief Excursion, *c*.1953, <http://www.bbc.co.uk/archive/nhs/5151.shtml>, accessed 29 January 2015.

27 J. H. Patterson, ‘Fathers At Delivery’, *British Medical Journal*, 1961, 1:5225, 594.

28 Interview with Alf/Mary (2014).

29 Helen Mathers and Tania McIntosh, *Born in Sheffield: A History of the Women's Health Services, 1864–2000* (Barnsley: Wharncliffe Books, 2000), 128.

30 Interview with Mary Croft, ‘Born in Sheffield’, 12.

31 Interview with Barbara Ford, ‘Born in Sheffield’, 13.

32 Interview with Henry (2013).

33 Interview with Mike (2013).

34 Elizabeth Roberts, *Women and Families: An Oral History, 1940–70* (Oxford: Blackwell, 1995), 98–9.

35 Interview with Glyn D, ‘A Man’s World’ collection, British Film Institute Archive, roll 56, 47–51.

36 Interview with Fred (2013). Also interviews with David S, ‘A Man’s World’, roll 88, 83–4; Frank D, ‘A Man’s World’, roll 82, 171; George R, ‘A Man’s World’, roll 134, 37–8; John C, ‘A Labour of Love’, British Library, roll 69, 1–3; Richard N, ‘A Man’s World’, roll 144, 8–9; Mr P6B, Elizabeth Roberts Archive (ERA) 1940–1970, 51.

37 Interview with Mary Lear (084), P. Thompson, and H. Newby, Families, Social Mobility and Ageing, an Intergenerational Approach, 1900–1988 [computer file]. Colchester, Essex: UK Data Archive (UKDA) [distributor], July 2005. SN: 4938, 88–9.

38 Interview with George Sadler (127), Families, 45; Interview with Alf/Mary (2014).

39 Interview with Ben (2013).

40 King, *Family Men*, 77–86; Szreter and Fisher, *Sex*, ch. 5.

41 King, *Family Men*, 174–84; Laura King, ‘“Now you see a great many men pushing their pram proudly”: Family-orientated Masculinity Represented and Experienced in Mid-twentieth-century Britain’, *Cultural and Social History*, 2013, 10, 599–617.

42 Interview with John C, ‘Labour of Love’, roll 69, 7.

43 Interview with Glyn D, ‘Man’s World’, roll 54, 20. Also interview with Alf, Steve Humphries and Pamela Gordon, *A Man's World: From Boyhood to Manhood, 1900–1960* (London: BBC Books, 1996), 170; Interviews with Hilda C, ‘Labour of Love’, roll 67, 5–6; Richard N, ‘Man’s World’, roll 144, 7; Sheila Barlow (004), Families, 45.

45 Woollett, White and Lyon, ‘Observations’, 73. They summarised evidence in recent published research and conference papers, including a study by Richards, Dunn and Antonis which found that 67 per cent of fathers were present in the Cambridge area in 1968–70, Oakley’s work, which found 74 per cent of men present in London in 1975–76, and White and Wollett, who reported 92 per cent attended in East End in 1980.

44 Interview with Henry (2013).

46 Colin Bell, Lorna McKee and Karen Priestley, *Fathers, Childbirth and Work: A Report of a Study* (Manchester: Equal Opportunities Commission, 1983), 29.

47 Brian Jackson, *Fatherhood* (London: George Allen and Unwin, 1984), 77.

48 Charlie Lewis, *Becoming a Father* (Milton Keynes: Open University Press, 1986), 62.

49 The total numbers are small but indicative. In 1970, 5/15 fathers attended the delivery of their children; in 1971 it was 4/12; 1972, 4/9; 1973, 6/13; 1974, 3/8; 1975, 5/7; 1976, 4/9; 1977, 4/5; 1978, 8/9; 1979, 8/12.

50 For example, men were on military service or incarcerated at the time of birth.

51 Interview with Meryl Thomas (2012).

52 Interview with Meryl Thomas (2012).

53 Interview with Cathy Warwick (2012). Liz Stephens recounted a similar story of the late 1970s, in which a man read his newspaper throughout, except to briefly tell his wife not to grunt. Interview with Liz Stephens (2012).

54 Interview with Cathy Warwick (2012).

55 Interview with Liz Stephens (2012).

56 Interview with Mavis Kirkham (2012). Also see Mavis Kirkham, ‘Basic Supportive Care in Labour: Interaction with and around Women in Labour’, (unpublished PhD thesis, University of Manchester, 1987), 61–3, 81. Sheena Byrom held a similar view. Interview with Sheena Byrom (2012).

57 Interview with Tom Smith, ‘Born in Sheffield’, 14.

58 Interview with Mary Croft, ‘Born in Sheffield’, 13.

59 Interview with Myra Snell (2012).

60 Interview with Sheila Duncan, ‘Born in Sheffield’, 11.

61 As Kirkham’s study shows, men often tried to please staff. Kirkham, ‘Basic Supportive Care’, 54.

62 Interview with Edward/Dorothy (2013).

63 Interview with Martin Byrne (020), Families, 30.

64 Interview with Michael Fell (057), Families, 62.

65 Interview with Gerald Handley (063), Families, 44.

66 Questionnaire respondent BQ44.

67 Interview with Peter Coverley (Jnr) (038), Families, 28. Also interviews with Elizabeth Arnold (002), Families, 97; John Dennis (049), Families, 41; Mrs J. Morris (093), Families, 35; Kathleen Murray (095), Families, 59; Harry Stainer (137), Families, 47–8; Trevor Welham (156), Families, 26; Arthur Winn (160), Families, 35. Questionnaire respondents BQ44, IM40, FD44, CR54, OJ55, CD71, IC68.

68 Interview with Robert Brotherston (018), Families, 48. Also interview with Elizabeth Arnold (002), Families, 97.

69 Interview with Edward/Dorothy (2013).

70 Interview with Barry (2013).

71 Interview with Leslie Lane (081), Families, 57. Also interviews with Alan Parks (106), Families, 48–9; Angela Ross (125), Families, 55–6; Michael Wall (150), Families, 34; Trevor Welham (156), Families, 25; Arthur Winn (160), Families, 35.

72 Catherine Niven, ‘How Helpful is the Presence of the Husband at Childbirth?’, *Journal of Reproductive and Infant Psychology*, 1985, 3, 45–53.

73 T. Dragonas, K. Thorpe and J. Golding, ‘Transition to Fatherhood: A Cross-cultural Comparison’, *Journal of Psychosomatic Obstetrics and Gynaecology*, 1992, 13, 5, 10.

74 Debbie Singh and Mary Newburn, *Becoming a Father: Men's Access to Information and Support about Pregnancy, Birth, and Life with a New Baby* (London: National Childbirth Trust, 2000), 49.

75 Hiranthi Jayaweera *et al*., ‘Pregnancy and Childbirth’, in Shirley Dex and Heather Joshi, eds, *Children of the 21st Century: From Birth to Nine Months* (Bristol: Policy Press, 2005), 124.

76 Interviews with Pat (2012); Sandra (2012); Sheena Byrom (2012).

77 Interview with Sheila Duncan, ‘Born in Sheffield’, 11.

78 McIntosh, *A Social History*, 153.

79 Interview with Mavis Kirkham (2012). Also see McIntosh, *A Social History*, 123.

80 For more on 1990s demonstrations, see McIntosh, *A Social History*, 131–4.

81 Interview with Cathy Warwick (2012).

82 Interview with Cathy Warwick (2012).

83 Interview with Meryl Thomas (2012).

84 Interview with Liz Stephens (2012).

85 Interview with Ali (2014).

86 Leavitt, *Make Room*, 287.

87 Linda Bryder, ‘Fathers and Hospital Childbirth in New Zealand’, *Social History of Medicine*, 2015, 28, 725–41.

88 Angela Brown, ‘Fathers in the Labour Ward: Medical and Lay Accounts’, in Lorna McKee and Margaret O’Brien, eds, *The Father Figure* (London: Tavistock Publications, 1982), 105.

89 Interview with Gary (2013).

90 Interview with Matt (2013).

91 Interviews with George (2014); James (2013); Jeremy (2014); Steve (2013); Gary (2013); Charlie (2013); Carl (2013).

92 Interview with Andrew (2013).

93 Interview with Bill (2013).

94 Interview with Aidan (2014).

95 For example, interviews with Carl (2013); Gary (2013); Mick (2013); Malcolm, (2013); questionnaire respondents OJ55, HL67, FZ75, MS74, RR78, UF81, KU79, QC81, UJ38, CR54, OI55. Also see Davis, ‘A Revolution’, 397.

96 Kirkham, ‘Basic Supportive Care’, 53.

97 For example, see Royal College of Midwives, ‘Reaching Out: Involving Fathers in Maternity Care’, 9, <https://www.rcm.org.uk/sites/default/files/Father’s%20Guides%20A4_3_0.pdf>, accessed 29 January 2015.

98 Interview with Dean (2014).

99 Interviews with Meryl Thomas (2012); Liz Stephens (2012).

100 Woollett, White and Lyon, ‘Observations’, 75.

101 Kirkham, ‘Basic Supportive Care’, 54.

102 Interview with Liz Stephens, (2012).

103 Interview with Cathy Warwick (2012).

104 On expectations and practices of fatherhood, see King, Family Men, ch. 3.

105 Tim Blackshaw, ‘Fathers and Childbirth’, in Caroline Squire, ed., *The Social Context of Birth* (Oxford: Radcliffe Publishing, 2004), 224.

106 Bryder, ‘Fathers’.

107 McIntosh, *A Social History*, 110–11; Interview with Mary Croft, ‘Born in Sheffield’, 12.

108 Questionnaire respondent ZB81. On changing norms of masculinity and fatherhood, see King, *Family Men*; Esther Dermott, *Intimate Fatherhood: A Sociological Analysis* (London: Routledge, 2008). On the connections between childbirth and masculinity, see Bryder, ‘Fathers’.

109 Richard Collier and Sally Sheldon, *Fragmenting Fatherhood: A Socio-legal Study* (Oxford: Bloomsbury, 2008), 61. See Leavitt, *Make Room*, 284.

110 Bryder, ‘Fathers’.

111 McIntosh, *A Social History*, 136–7.

112 Interviews with Pat (2012); Liz Stephens (2012).

113 Interview with Mavis Kirkham (2012).

114 Interviews with Pat (2012); Liz Stephens (2012); Meryl Thomas (2012).

115 This was highlighted particularly by Mavis Kirkham. Interview with Mavis Kirkham (2012).

116 For example, see ‘Fathers in delivery room could make pain of childbirth worse, study suggests’, *Telegraph*, 21 January 2015, <http://www.telegraph.co.uk/news/science/science-news/11357662/Fathers-in-delivery-room-could-make-pain-of-childbirth-worse-study-suggests.html>, accessed 29 Janaury 2015. Pat, Liz and Myra highlighted the pressure towards men’s presence, and the possibility that other birth partners might be better, whilst Sandra suggested that staff assumed there was a problem or specific reason if men did not attend appointments or the labour with their partners. Interviews with Pat (2012); Liz Stephens (2012); Myra Snell (2012); Sandra (2012).

